# Cytotoxicity and anti-tumor effects of new ruthenium complexes on triple negative breast cancer cells

**DOI:** 10.1371/journal.pone.0183275

**Published:** 2017-09-12

**Authors:** Cecília P. Popolin, João P. B. Reis, Amanda B. Becceneri, Angélica E. Graminha, Márcio A. P. Almeida, Rodrigo S. Corrêa, Legna A. Colina-Vegas, Javier Ellena, Alzir A. Batista, Márcia R. Cominetti

**Affiliations:** 1 Departmento de Gerontologia, Universidade Federal de São Carlos, São Carlos, São Paulo, Brazil; 2 Departmento de Química, Universidade Federal de São Carlos, São Carlos, São Paulo, Brazil; 3 Departamento de Química, Universidade Federal do Maranhão, Bacanga, São Luís, Maranhão, Brazil; 4 Departamento de Química, Universidade Federal de Ouro Preto, Morro do Cruzeiro, Ouro Preto, Minas Gerais, Brazil; 5 Departamento de Física e Ciência Interdisciplinar, Instituto de Física de São Carlos, Universidade de São Paulo, São Carlos, São Paulo, Brazil; University of South Alabama Mitchell Cancer Institute, UNITED STATES

## Abstract

Triple-negative breast cancer (TNBC) is a highly aggressive breast cancer subtype. The high rate of metastasis associated to the fact that these cells frequently display multidrug resistance, make the treatment of metastatic disease difficult. Development of antitumor metal-based drugs was started with the discovery of cisplatin, however, the severe side effects represent a limitation for its clinical use. Ruthenium (Ru) complexes with different ligands have been successfully studied as prospective antitumor drugs. In this work, we demonstrated the activity of a series of biphosphine bipyridine Ru complexes **(1)** [Ru(SO_4_)(dppb)(bipy)], **(2)** [Ru(CO_3_)(dppb)(bipy)], **(3)** [Ru(C_2_O_4_)(dppb)(bipy)] and **(4)** [Ru(CH_3_CO_2_)(dppb)(bipy)]PF_6_ [where dppb = 1,4-bis(diphenylphosphino)butane and bipy = 2,2’-bipyridine], on proliferation of TNBC (MDA-MB-231), estrogen-dependent breast tumor cells (MCF-7) and a non-tumor breast cell line (MCF-10A). Complex **(4)** was most effective among the complexes and was selected to be further investigated on effects on tumor cell adhesion, migration, invasion and in apoptosis. Moreover, DNA and HSA binding properties of this complex were also investigated. Results show that complex **(4)** was more efficient inhibiting proliferation of MDA-MB-231 cells over non-tumor cells. In addition, complex **(4)** was able to inhibit MDA-MB231 cells adhesion, migration and invasion and to induce apoptosis and inhibit MMP-9 secretion in TNBC cells. Complex **(4)** should be further investigated *in vivo* in order to stablish its potential to improve breast cancer treatment.

## Introduction

Breast cancer is the most prevalent type of cancer in women and the second leading cause of cancer death worldwide [1]. Chemotherapy is one of the most extensively methods used to treat metastasis from many types of cancer. However, its efficacy and safety remain a primary concern as well as its toxicity and other side effects. Moreover, the development of chemotherapy resistance is a major obstacle to the effective treatment of many tumors, including breast cancer [2]. Triple negative breast cancer (TNBC), in which cells do not have estrogen (ER-), progesterone (PR-), and HER2 (HER2-) receptors is a highly aggressive breast cancer subtype, responsible for about 20% of breast cancers. The high rates of metastasis associated to the fact that these cells frequently display multidrug resistance make the treatment of its metastatic disease difficult [3, 4]. TNBC is treated with a combination of therapies such as surgery, radiation, and chemotherapy. However, the limited efficacy of current systemic and targeted therapies against TNBC tumor metastases leads the search for new types of treatments [5].

Cisplatin, oxaliplatin and carboplatin are the only metal-based chemotherapeutic drugs approved for worldwide clinical practice. They have been used and are effective for the treatment of numerous human cancers. However, cisplatin has been reported to cause drug resistance and several undesirable side effects such as allergic reactions, decrease immunity to infections, severe kidney problems, gastrointestinal disorders, haemorrhage, and hearing loss [6]. Ru complexes have emerged as potential candidates to replace platinum chemotherapy. The Ru complex, known as NAMI-A (imidazolium *trans*-[tetrachloro(dimethylsulfoxide)(1H-imidazole)ruthenate(III)]), which was proven to be effective in managing lung cancer metastases is in clinical phase of studies. Currently, a combinatory therapy using NAMI-A and gemcitabine is in Phase I/II of clinical trial for the treatment of non-small cell lung carcinoma [7]. The KP1019 (*trans*-[tetrachlorobis(1H-indazole)-ruthenate(III)]), is a second Ru-based anticancer agent to enter to clinical trials and the results of the early clinical development demonstrated that five out of six evaluated patients experienced disease stabilization with no severe side effects [8].

Over the last years, some unique properties of Ru-based complexes were identified, including the possibility to occupy a high number of spatial positions compared to cisplatin, a high number of potential accessory molecules that can be carried by the drug structure and particularly, the possibility to exist in the biological fluids in almost all the most important oxidation states from II to IV [9]. These characteristics have made Ru-based complexes promising antitumor and antimetastatic candidates. Nevertheless, the underlying mechanisms of the antimetastatic potential of Ru-based agents are not completely understood. Thus, in this work we described the effects of a series of Ru complexes **(1)** [Ru(SO_4_)(dppb)(bipy)], **(2)** [Ru(CO_3_)(dppb)(bipy)], **(3)** [Ru(C_2_O_4_)(dppb)(bipy)] and **(4)** [Ru(CH_3_CO_2_)(dppb)(bipy)]PF_6_ [were dppb = 1,4-bis(diphenylphosphino)butane and bipy = 2,2’-bipyridine] on the proliferation of TNBC (MDA-MB-231), ER+ (MCF-7) breast tumor cells and non-tumor breast cell line (MCF-10A). These complexes were inspired by the platinum-based anticancer drugs carboplatin, oxaliplatin, nedaplatin and lobaplatin, which have oxygen containing ligands in their structures and because the facility of their syntheses, in high yield, with high purity. Also, the complex **(4)** was introduced in this series mainly because it is a cationic complex, which could be more soluble in the biological medium.

Therefore, the aim of this work was to investigate the effects of the newly synthesized Ru complexes on different types of breast tumor cells and, after identifying the more cytotoxic complex, to further investigate its effects on cell adhesion, migration, invasion, induction of apoptosis and interaction with DNA and HSA in TNBC cells. Complex **(4)** demonstrated the lowest IC_50_ inhibiting MDA-MB-231 cell proliferation and also, to be effective inhibiting cell adhesion, migration and invasion and inducing apoptosis of this TNBC cell line. Complex **(4)** was able to bind DNA and HSA with low to moderate intensity, but even so, it was capable to induce conformational changes on the DNA molecule.

## Materials and methods

**Fig 1:**
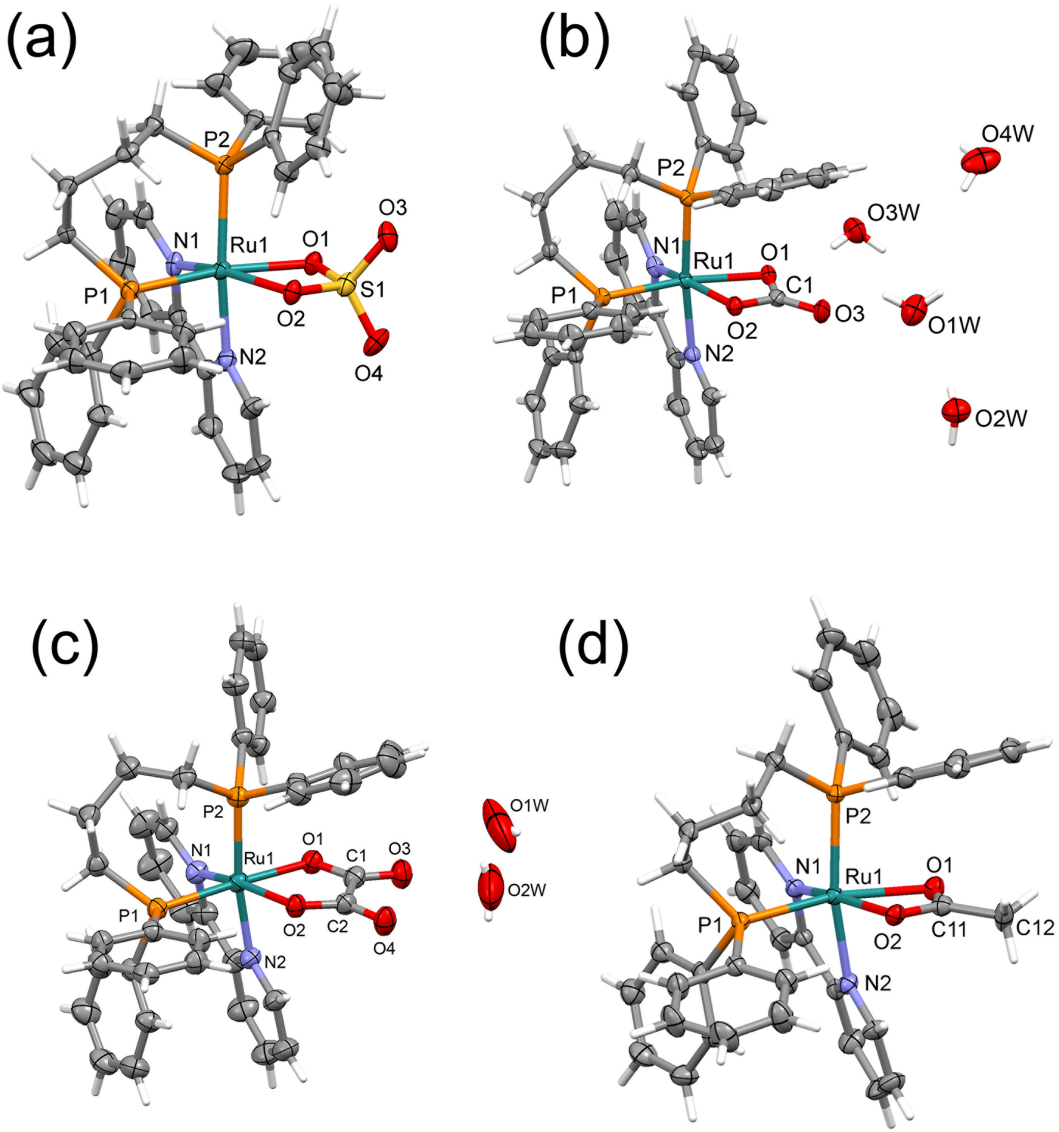
Crystal structures of the complexes. **(A) (1)** [Ru(SO_4_)(dppb)(bipy)], **(B) (2)** [Ru(CO_3_)(dppb)(bipy)], **(C) (3)** [Ru(C_2_O_4_)(dppb)(bipy)] and **(D) (4)** [Ru(CH_3_CO_2_)(dppb)(bipy)]PF_6_, showing the atoms labelling and the 30% probability ellipsoids. For 4, the PF_6_^-^ anion was omitted, for sake of clarity.

All nuclear magnetic resonance (NMR) experiments were recorded on a BRUKER spectrometer, in a BBO 5 mm probe at room temperature, observing ^31^P proton-decoupled at 161.98 MHz in CH_2_Cl_2_ using a capillary of D_2_O. In the ^31^P{^1^H} experiments chemical shifts are with respect 85% H_3_PO_4_ signal as external reference. The electronic spectra were obtained through a scanning in a Hewlett–Packard diode array, model 8452A, spectrophotometer. The elemental analyses were performed using a FISONS CHNS, mod. EA 1108 micro analyzer. Crystals of **(1–4)** complexes were grown by slow evaporation of a dichloromethane/diethyl ether solution (2:1), at room temperature. X-ray diffraction experiments were carried out using a suitable crystal mounted on glass fiber, and positioned on the goniometer head. The data collection were performed using Mo-Kα radiation (λ = 0.71073 Å) on an Enraf-Nonius Kappa-CCD diffractometer. Data were collected with the COLLECT program. Data reduction was carried out using the software Denzo-Scalepack software. Gaussian or semi-empirical corrections from equivalents absorption were carried out. The structures were solved by the Direct method using SHELXS-97 and refined using the software SHELXL-97. The models were refined by full-matrix least squares on F^2^ by means of SHELXL-97. The hydrogen atoms were calculated at idealized positions using the riding model option of SHELXL97. The S2 Table summarizes main crystallographic data for the complexes **(1–4)**. The ORTEPs showed in Fig 1 were prepared with ORTEP-3 for Windows.

### Preparation of the complexes

The precursor [RuCl_2_(dppb)(bipy)] was prepared as previously described. [10] The salts Na_2_SO_4_, Na_2_CO_3_, Na_2_C_2_O_4_, and CH_3_COONa were purchased from Sigma-Aldrich. All manipulations were carried out under purified argon using the standard Schlenk technique. Complexes **(1–3)**: In a typical synthesis, 0.59 g (0.66 mmol) of the precursor, *cis*-[RuCl_2_(dppb)(bipy)] was dissolved in 10 mL of methanol and 0.80 mmol of the salt [Na_2_SO_4_ (0.11 g), Na_2_CO_3_ (0.084 g), Na_2_C_2_O_4_, (0.107 g)], was dissolved in the minimum quantity of water, which was added drop wise to the methanolic solution. The mixture, under stirring, was heated at reflux, for 4 h, after what it was evaporated until to about 2 mL, and ether was added to precipitate a solid product, which was filtered off, washed with water (3 x 5 mL) and ether (3 x 5mL), and dried in vacuum (S1 Fig). Elemental analyses for **(1)**: C_38_H_36_N_2_O_4_P_2_SRu.2H_2_O exp. (calc) C% 55.72 (55.95), H% 4.83 (4.94), N% 3.23 (3.43); for **(2)**: C_39_H_36_N_2_O_3_P_2_Ru.4H_2_O, C%, 57.33 (57.42), H%, 5.28 (5.43), N%, 3.67 (3.43); for **(3)**: C_40_H_36_N_2_O_4_P_2_Ru.2H_2_O, C%, 59.71 (59.48), H%, 4.91 (4.99), N%, 3.59 (3.47). ^31^P{^1^H} NMR data for **(1)**, 50.0 (d) and 34.6 (d), J = 43.5 Hz; for **(2)**, 47.2 (d) and 46.1 (d), J = 33.3 Hz; for **(3)**, 48.4 (d) and 47.3 (d), J = 33.2 Hz. Complex **(4)**: For the preparation of the complex [Ru(CH_3_CO_2_)(dppb)(bipy)]PF_6_ the same above procedure was adopted, but in this case 0.065 g (0.8 mmol) of the salt CH_3_COONa, was used. Thus, after the reflux of the solution for 4 h, 0.13 g (0.80 mmol) of NH_4_PF_6_ was added and the reflux was left 1 h longer, afterwards, the solid precipitated was filtrated off and washed with water (3 x 5mL) and dried in vacuum. Elemental analysis for **(4)**: C_40_H_39_N_2_O_2_F_6_P_3_Ru, expl. (calc) C%, 54.31 (54.12), H%, 4.50 (4.42), 3.12 (3.16). ^31^P{^1^H} NMR, in CH_2_Cl_2_; D_2_O 48.7 (d) and 47.3 (d), J = 33.4 Hz.

### Cell lines and culture

MDA-MB-231 human breast tumor cells and MCF7 human breast tumor cells, obtained from Rio de Janeiro Cell Bank, were maintained at 37°C in 5% CO_2_ in DMEM medium containing fetal bovine serum (FBS) 10% (Vitrocell). MCF-10A, a non-tumor breast cell line, obtained from Rio de Janeiro Cell Bank, was cultivated in DMEM/F12 medium containing horse serum (HS) 5%, EGF (0.02 mg/mL); hydrocortisone (0.05 mg/mL); cholera toxin (0.001 mg/mL) and insulin (0.01 mg/mL). Both media contained penicillin (100 UI/mL), streptomycin (100 mg/mL) and L-glutamine (2 mM).

### Cell morphology

Exponentially growing MDA-MB-231, MCF-7 or MCF-10A cells were harvested, counted and seeded (1x10^5^ cells/mL) and plated on sterile 12-well plates for 24 h. Cells were allowed to grow at 37°C in 5% CO_2_ overnight and then, treated or not (control) with different concentrations of the complexes **(1–4)** for 2 and 24 h. Cell morphology was examined under an inverted microscope (Nikon, T5100) with amplification of 100×.

### Cell proliferation

Proliferation assays were performed as described earlier [11]. Briefly, cells lines were prepared in a concentration of 1x10^4^ cells/100 μL, in complete medium (with 10% FBS), and plated on sterile 96-well plates for 24 h. The culture medium was removed from the wells and a new one, supplemented with 10% FBS and containing different concentrations of **(1)** [Ru(SO_4_)(dppb)(bipy)] **(2)** [Ru(CO_3_)(dppb)(bipy)], **(3)**, [Ru(C_2_O_4_)(dppb)(bipy)], or **(4)** [Ru(CH_3_CO_2_)(dppb)(bipy)]PF_6_, was added to the wells. Cells were incubated for 24 h under the same conditions as described above. Cell proliferation assay was performed in comparison with untreated control. After incubation, the culture medium of each well was removed and a solution containing MTT (1 mg/mL) was added (100 μL/well). The plates were then kept at 37°C for 4 h and the formed crystals were dissolved in DMSO. The absorbance was read on an ELISA plate reader at a wavelength of 540 nm. Cisplatin was used as a positive control for cell proliferation inhibition. The selectivity index (SI) was calculated as the ratio IC_50_ (MCF-10A) / IC_50_ (MDA-MB-231).

### Colony formation

Exponentially growing MDA-MB-231 cells were harvested, counted and seeded (300 cells/plate) into Petri dishes. Cells were allowed to grow at 37°C in 5% CO_2_ overnight and then, treated with different concentrations of complex **(4)** for 3 h. After this time, the medium was changed to a fresh medium without any complex. After incubation for additional 10 days the cells were rinsed with PBS, fixed with methanol and acid acetic 3:1 for 5 min and stained with methanol and crystal violet 5% for 15min. Relative survival was calculated from the number of single cells that formed colonies of >50 cells on the tenth day.

### Migration

The effects of complex **(4)** on tumor cell migration was investigated by two methods, wound healing and transwell assays using Boyden chambers. For wound healing, MDA-MB-231 cells (2 x10^5^/mL) were plated in 12-wells plates and incubated properly until the culture reached 100% of confluence. Afterwards, a straight scratch was made with a sterile pipette tip and cells were washed with culture medium to remove unbound cells and debris. Cells were incubated with complex **(4)** 5, 10 and 20 μM for 24 and 48 h. Cells were viewed using an inverted microscope (Nikon, T5100) at 40× total magnification and images were captured (Moticam, 1000-S camera) at 0 h, 24 h and 48 h. Closure area of migrating cells was measured using Image J software, and the percentage of wound closure was calculated, comparing time zero and 48 h, using a formula from Yue and co-workers [12].

$$\%\;wound\;closure=\frac{\left(A_{t=0h}-A_{t=\Delta h}\right)}{\left(A_{t=0h}\right)}\text{x }\left(100\right)$$

Cell migration was also assessed in a chemotactic assay using 24 well Boyden chambers (BD Biosciences) as described earlier [13]. MDA-MB-231 (0.5 x 10^5^/350 μL) cells, incubated or not with complex **(4)** 5, 10 and 20 μM were seeded on the upper chamber in a DMEM incomplete medium (without FBS). In the lower chamber DMEM medium supplemented with 10% FBS was added. Cells were allowed to migrate for 22 h at 37°C and 5% CO_2_ in a humidified environment. Then, cells that remained in the upper chamber were removed using a cotton swab. Cells that migrated to the other side of the upper chamber membrane were fixed with methanol and stained with 1% toluidine blue. Migrated cells were quantified by manual counting and inhibition ratio was expressed as % of control.

### Invasion

The effect of different concentrations of complex **(4)** 5, 10 μM and 20 μM on MDA-MB-231 tumor cell invasiveness was determined by the ability to transmigrate through a layer of matrigel in a transwell chamber (BD Biosciences) as described earlier [14]. Wells were rehydrated with the addition of 1mL of warm (37°C) incomplete culture medium for 2 h. MDA-MB-231 cells (0.5 x 10^5^/350 μL) were loaded on the top of matrigel in a DMEM incomplete medium. DMEM medium supplemented with 10% FBS was placed on the bottom chamber of the transwell units. Twenty-two hours later, cells that remained in the upper chamber were removed using a cotton swab and invasive cells were fixed with methanol and stained with 1% toluidine blue. The invaded cells were quantified by manual counting and inhibition ratio was expressed as % of control.

### Zymography

MDA-MB-231 cells (1.0 x 10^5^/well) were seeded into 12-wells culture plates and cultured in a medium containing FBS 10% to near confluence (80%) of the cell monolayer. Monolayers were carefully wounded using a pipette tip, and any cellular debris present was removed by washing with PBS. The wounded monolayers were then incubated in serum-free medium containing 2.5, 5, 10 and 20 μM of complex **(4)** for 24 h. After this time, supernatants of the wound healing assay were collected and tested for MMP secretion as previously described [15]. Briefly, equal amounts of total protein (10 mg/lane) were subjected to electrophoresis. Zymography gels consisted of 10% polyacrylamide impregnated with gelatin at a final concentration of 1% in the presence of sodium dodecyl sulfate (SDS) under non-reducing conditions. After 2 h of electrophoresis (70 mV), the gels were washed twice for 20 min in a 2.5% Triton X-100 solution, and incubated at 37°C for 20 h in a substrate buffer (50mM Tris–HCl, pH 8.5, 5 mM CaCl_2_ and 0.02% NaN_3_). Gels were then stained with coomassie brilliant blue for 30 min and destained in methanol and acetic acid for 20 min. Gelatin degrading enzymes were visualized as clear white bands against a blue background, indicating proteolysis of the substrate protein. The molecular mass of gelatinolytic activities was determined by comparison to reference protein molecular mass marker PageRuler Prestained Protein Ladder (Thermo Fisher Scientific). Activity bands were identified following the previous description according to their molecular weights.

### Adhesion

The effects of complex **(4)** on the adhesion of MDA-MB-231 cells were analyzed in 96-well plates (Corning) as described earlier [16]. Vitronectin (1.0 μg), laminin (0.3 μg) or fibronectin (0.3 μg) immobilized on the plates in a cell adhesion buffer (20 mM HEPES, 150 mM NaCl, 5 mM KCl, 1 mM MgSO_4_ and 1 mM MnCl_2_ pH 7.35), overnight at 4°C. Collagen type I (10 μg) was dissolved in acetic acid (0.1%) and coated on the wells. On the next day, wells were blocked with adhesion buffer containing 1% BSA solution for 1h and then washed with 100 μL per well of adhesion buffer. Cells (5 x 10^4^/100 μL) were incubated for 30 min with different concentrations of the complex **(4)** at 37°C 5% CO_2_ and then plated and incubated under the same conditions for a further 1h. Then the non-adhered cells were gently removed by washing and attached cells were fixed with 100 μL 70% ethanol for 10 min. Cells were stained with 0.5% crystal violet for 20min and the excess dye was removed by washing with PBS. The stained cells were diluted in 100 μL of 1% solution of sodium dodecyl sulfate (SDS) for 30min and reading of the plates was performed on ELISA reader at 595 nm wavelength.

### Phalloidin staining

To examine the effects of complex **(4)** on the F-actin cytoskeleton organization in MDA-MB-231 cells, Alexa Fluor^®^ 488 Phalloidin was used to stain the F-actin fibers. MDA-MB-231 cells (5x10^4^ cells/100 μL) were plated in 96-well plates and maintained at 37°C in a humidified incubator with 5% CO_2_ for 24 h. In the next day, cells were treated with 10, 20, 40 μM of complex **(4)** and incubated for 30 min. Next, cells were washed with PBS, fixed with 3.7% paraformaldehyde in PBS for 30 min and then permeabilized with 0.1% Triton-X 100 in PBS for 5min at room temperature. Blocking was performed with 2% BSA for 30 min, followed by the addition of Alexa Fluor 488 Phalloidin for 20 min. Cells were then stained with DAPI for nuclear labeling for 10min and finally washed again with PBS for three times. Images were obtained with an automated microscope ImageXpress^®^ Micro XLS System (Molecular Devices).

### Apoptosis

#### DAPI staining

The apoptotic activity of the complex **(4)** was analyzed by DAPI staining and flow cytometry with PE-Annexin-V Apoptosis Detection Kit (BD Biosciences). For DAPI staining, MDA-MB-231 cells (2.0 x 10^4^/100 μL) were seeded in 96-wells plates and maintained at 37°C in a humidified incubator with 5% CO_2_ for 24 h. In the next day, cells were treated with 60 and 70 μM of complex **(4)** and incubated for 3 h. Next, cells were washed with PBS, fixed with methanol and stained with DAPI 1 μg/mL (Life Technologies, Carlsbad, CA) in DMEM medium without FBS for 10 min. Fluorescence was captured in automated microscope ImageXpress^®^ Micro XLS System (Molecular Devices).

#### Flow cytometry

MDA-MB-231 and MCF-10A cells (1.0 x 10^5^/mL) were seeded in 12-well plates in a complete DMEM medium and incubated for 24 h. After this period the medium was removed and cells were incubated or not (control) with different concentrations of complex **(4)**, for 3 h at 37°C and 5% CO_2_. After treatment the plate was centrifuged at 2000 rpm for 5 min at 4°C, washed with PBS and resuspended in 400 μL of binding buffer from the kit. Cells were incubated with 10 μL of 7AAD and 10 μL of PE-Annexin-V for 15 min protected from light. After the incubation period the supernatant was removed and 400 μL of binding buffer were added to the wells. Cells were then removed from the wells with the aid of a scraper and transferred to flow cytometry tubes. The reading was performed in Accuri C6 flow cytometer (BD Biosciences) and fluorescence emitted by each dye was quantified using CellQuest software (BD Biosciences).

### Reverse Transcriptase Quantitative Real Time PCR (RT-qPCR)

MDA-MB-231 cells (1.0 x 10^6^/plate) were incubated for 3 h with (20 and 40 μM) or without (control) the complex **(4)** in Petri dishes (6 cm) at 37°C in a humidified incubator with 5% CO_2_. Total RNA was extracted using Trizol reagent (Invitrogen). cDNAs were synthesized using Enhanced Avian RT First Strand Synthesis kit (Sigma-Aldrich, St. Louis, MO, USA). A Rotor-Gene 6000 real-time rotary analyzer (Corbett Life Science, Australia) was used to amplify both target and internal control templates (1 cycle at 95°C for 5 min and 40 amplification cycles at 95°C for 30 sec, 55°C for 30sec and 72°C for 45sec). In brief, 1 μL of reverse transcribed product template, 5 μL of SYBR Green JumpStart Taq ReadyMix (Sigma-Aldrich) and the gene-specific primer pairs at a final concentration of 500 nmol l^-1^ for each primer, made 10 μL of reaction system. Primers used in the assays were: Caspase-3 (Forward: 5’GTG CTA CAA TGC CCC TGG AT3’; Reverse: 5’GCC CAT TCA TTT ATT GCT TTC C3’), Bax (Forward: 5’CAT CCA GGA TCG AGC AGG3’; Reverse: 5’CGA TGC GCT TGA GAC ACT C3), Bcl-2 (Forward: 5’GGT GGG AGG GAG GAA GAA T3’; Reverse: 5’GCA GAG GCA TCA CAT CGA C3’) and β-actin (Forward: 5’GAC GGC CAG GTC ATC ACC ATT G3’; Reverse: 5’AGC ACT GTG TTG GCG TAC AGG 3’). Bax primers (NM_001291428.1) and Bcl-2 (NM_000633.2) were designed with Gene Runner available at http://generunner.net/ (version5.0.63 Beta), except for the primer Caspase-3 (NM_004346.3). For each gene, all samples were amplified simultaneously in duplicate in one assay run. The internal calibrator used as a basis to standardize the results of expression was the control group ΔCts average. Calibration was determined by ΔΔCt = ΔCt (sample) − ΔCt (calibrator). Gene expression was assessed by relative quantification, using the formula 2-ΔΔCt [17] and β-actin as internal control [18]. Data represent three assays in duplicate. A blank with water, primers and SYBR Green instead of template sample was performed.

### Western blotting

MDA-MB-231 cells (1.0 x 10^6^/plate) were incubated for 6 h with (2.5, 5 and 10μM) or without (control) the complex **(4)** in Petri dishes (6 cm) at 37°C in a humidified incubator with 5% CO_2_. After incubation, cells were lysed using RIPA buffer (150 mM NaCl; 50 mM Tris-HCl pH 7.4; 50 mM NaF; 2 mM EDTA; 1.0% NP-40; 0.5 mM Na-deoxycholate; 0.1% SDS, pH 8.0) and the content was transferred to a 1.5 mL microtube. Protein concentrations of supernatants were determined using DC Protein Assay kit (Bio-Rad). Protein samples (15.0 μg) were applied onto a 4–20% Tris-glycine gels, transferred to nitrocellulose membranes (BioRad Laboratories) and incubated with anti-Caspase-3, anti-Caspase-9 (inactive and cleaved forms), and anti-Bcl-2 antibodies (1:1000) (BD Biosciences), followed by incubation with HRP-conjugated goat anti-mouse secondary antibody (1:5000) (Thermo Scientific). Beta-actin was used as endogenous control. Substrate development was performed using SuperSignal West Dura Extended Substrate reagent (Thermo Scientific). Specific bands were visualized with ChemiDoc MP imager (BioRad Laboratories) and quantified with Image J software, normalized to β-actin.

### Partition coefficient *(P)*

The water-octanol partition coefficients were determined by use of the stir-flask method [19]. The complex was added in a mixture with equal volumes of water and octanol shaking by 24 h at 100 rpm and 37°C, samples were centrifuged for 5min at 300rpm, the organic and aqueous phases were separated. The concentration of drug in each phase was measured spectrophotometrically in order to determine values of *P* = [drug] (in octanol)/[drug] (in water).

### Interaction studies with HSA

For fluorescence measurements, the HSA concentration in Tris–HCl buffer was kept constant in all the samples, while the complex concentration was increased from 0.50 to 50 μM, and quenching of the emission intensity of the HSA tryptophan residues at 305 nm (excitation wavelength 270 nm) was monitored at different temperatures (25°C and 37°C). The experiments were carried out in triplicate and analysed using the classical Stern-Volmer equation. The binding constant (K_b_) and number of complexes bound to HSA (n) were determined by plotting the double log graph of the fluorescence data using: log [(F_0_-F)/F] = log K_b_ + nlog[Q]. The thermodynamic parameter (ΔH) was calculated from equation: ln (K_2_/K_1_) = [(1/T_1_)-(1/T_2_)]ΔH/R, where K_1_ and K_2_ are the binding constants at temperatures T_1_ and T_2_, and R is the gas constant. Furthermore, the change in free energy (ΔG) and entropy (ΔS) were calculated from the following equation: ΔG = -RT lnK = ΔH − TΔS.

### Spectroscopic measurements

All measurements with *ct-*DNA (calf thymus DNA) were carried out in Tris-HCl buffer. The spectroscopic titrations were carried out by adding increasing amounts of DNA to a solution of the complex, recording the UV-Vis spectrum. The intrinsic binding constant Kb was determined from the plot of [DNA]/(εa-εf) *vs* [DNA], using the neighbour exclusion equation.

### Circular dichroism experiments

CD spectra were recorded on a spectropolarimeter JASCO J720 between 400 and 240nm in a continuous scanning mode (200 nm/min). All CD spectra were generated and represented averages of three scans. An appropriate volume of each solution was added to the samples of a freshly prepared solution of *ct-*DNA (100 μM) to achieve molar ratios ranging from 0.06 to 0.50 drug/DNA. The samples were incubated at 37°C for 18 h.

### Agarose gel electrophoresis studies

Ten microliters of pBR322 plasmid DNA in a Tris-HCl buffer were incubated at 37°C for 18 h with molar ratios between 0.12 and 0.5. After incubation, 5 μL of each sample was separated by electrophoresis in a 1% agarose gel for 45 min at 100V using a Tris borate–EDTA buffer (TBE) and stained with ethidium bromide (2 mL ethidium bromide per 40 mL agarose gel mixture). Samples of DNA with DMSO were used as controls. The DNA bands were visualized as an image using a UV light transilluminator (ChemiDoc MP, BioRad Laboratories).

### Statistical analysis

Each experiment was repeated three times in triplicate and a standard error mean was calculated. Shapiro-Wilk’s test was used to verify data normality. As normal distribution was present, the results were compared statistically with a one-way or two-way analysis of variance (ANOVA). Since the ANOVA tests showed significant differences (acceptable *p* level < 0.05), Bonferroni’s significant difference post hoc analyses were performed to determine differences between simple and grouped main-effect means, respectively. The data were analysed and IC_50_ calculations were made using Hill’s equation in the GraphPad Prism software (version 6.05).

## Results and discussion

### Synthesis

The complexes **(1–4)** were synthesized from the precursor *cis*-[RuCl_2_(dppb)(bipy)], reacting with the respective salts Na_2_SO_4_, Na_2_CO_3_, Na_2_C_2_O_4_, and CH_3_COONa, obtaining products with high yield and purity (S1 Fig). The complexes were characterized by elemental analysis and ^31^P{^1^H} NMR. Also, ^31^P{^1^H} NMR experiments, at 37°C, showed that the four new complexes are stable, in DMSO solution, for at least 48 h. Crystal structures of the complexes **1–4** were determined by single-crystal X-ray diffraction (Fig 1).

The Ru-N and Ru-P bond lengths for the complexes (S1 Table) are in the expected range found for similar ruthenium/phosphine/diimine complexes [20]. The S-O and S = O bond lengths for complex **(1)**, at ~1.52 and ~1.45 Å, respectively, are in the expected range found in the literature, when this group is coordinated to transition metal. The C = O bond lengths for complexes **(2)**, **(3)** and **(4)** are close to 1.27 Å, as found for [Ru(amino acid)(bipy)(dppb)]PF_6_ (amino acid = glycine and leucine) complexes, which are coordinated to the ruthenium center by the nitrogen and by the carboxylate group [21]. It is interesting to compare that the Ru-O(1) bond lengths are longer than Ru-O(2) distances, for all studied complexes. This behaviour is a consequence of the strong *trans* effect of the phosphorus atoms, which is stronger than the *trans* effect of the nitrogen of the pyridine ring.

### Cell morphology and proliferation

**Fig 2:**
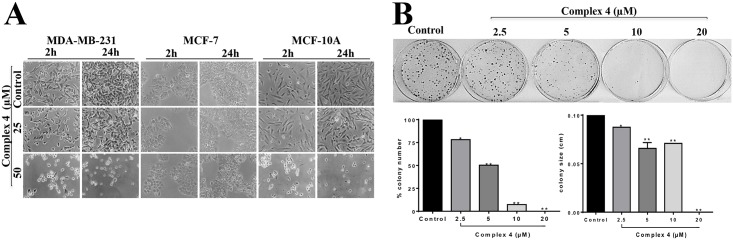
Effect of complex 4 on MDA-MB-231, MCF-7 and MCF-10A cells. **(A)** Cell morphology was examined after 2 h and 24 h of treatment. **(B)** Clonogenic assay of untreated MDA-MB-231 cells (control) or cells treated with complex **(4)**. A photograph of a representative experiment is shown along with graph quantifications of colony number and size. Significant at the *p<0.005, **p<0.001 levels using ANOVA and Bonferroni tests.

In this work the cytotoxicity of the complexes was evaluated against the MDA-MB-231, MCF-7 and MCF-10A cells by morphology, clonogenic and MTT assays (Fig 2, Table 1). To investigate the effects on cell morphology, the complexes were incubated with cells at concentrations of 25 and 50 μM for 24 h. Complex **(1)** did not alter the morphology of MDA-MB-231 and MCF-7 cells, when compared to untreated cells. However, complex **(1)** at both concentrations tested, altered the morphology of MCF-10A cells besides provoking a decrease in cell density, as compared to untreated cells (S2 Fig). Complex **(2)** at 50 μM incubated with MDA-MB-231 cells profoundly altered their morphology, promoting the appearing of round cells, which is an indicative of cell detachment and death. Complex **(2)** did not alter the morphology of MCF-10A or MCF-7 cells (S3 Fig). Complex **(3)** incubated with MCF-10A cells at concentration of 50 μM reduced cell density, but did not alter the morphology of MDA-MB-231 and MCF-7 cells (S4 Fig). The incubation of the complex **(4)** with MDA-MB-231 and MCF-10A cells, at concentration of 50 μM profoundly altered their morphology, promoting the appearing of round cells, also indicating cell death. The incubation of this complex with MCF-7 cells was not effective altering morphology (Fig 2A).

**Table 1:** IC_50_, SI and Log *P* values for complexes (1–4) and cisplatin in each cell line, after 24 h of incubation.

Complex	IC_50_ ±SEM (μM)				
	MDA-MB-231	MCF-10A	MCF-7	^a^SI	Log *P*
Precursor	>200	81.87 ±7.87	>200	0.41	-0.40 ± 0.01
(1)	>200	42.75 ± 5.02	>200	0.21	0.26 ± 0.06
(2)	52.74 ± 0.62	31.46 ± 1.06	>200	0.60	0.55 ± 0.02
(3)	>200	42.18 ± 2.37	>200	0.21	0.06 ± 0.008
(4)	31.16 ± 0.04	48.89 ± 0.09	>200	1.57	0.77 ± 0.03
Cisplatin	2.43±0.20	29.45 ± 0.85	79.18 ± 0.20	12.20	-2.53^b^

Precursor: *cis*-[RuCl_2_(dppb)(bipy)]. SEM = Standard error of the mean.

^a^(SI) Selectivity index of the compounds are calculated as described in Methods section.

^b^Log *P* of cisplatin was based on Buss *et al*. [22].

MTT assays demonstrated that all four complexes are not active against the MCF-7 tumor cells (Table 1). Since complex **(4)** exhibited the lowest IC_50_ (31.16 ± 0.04 μM) and the highest selectivity index (1.57) on the proliferation of MDA-MB-231 TNBC cells, it was selected to be further investigated on its biological mechanism of action. In agreement with our results, Wu and colleagues [9] described that the arene ruthenium(II) complex inhibits more effectively the proliferation of MDA-MB-231 cells (IC_50_ 20.8 μM) compared to non-tumor MCF-10A cell (IC_50_ > 300 μM), and human normal kidney HK2 cells (IC_50_ 110.3 μM). This could indicate specificity of the complexes, to affect tumor cells, but not normal cells’ viability.

Colony formation assay is an *in vitro* cell survival assay based on the ability of a single cell to grow into a colony. This is a method of choice to determine cell reproductive death after treatment with ionizing radiation, but can also be used to determine the effectiveness of other cytotoxic agents [23]. The results show that the complex **(4)** at concentrations of 2.5, 5, 10 and 20 μM significantly inhibited the colony number and size in MDA-MB-231 cells, compared to untreated control cells, acting therefore as a cytotoxic and cytostatic agent. The highest concentration (20 μM) completely abolished the capacity of breast tumor cells to form colonies (Fig 2B).

These findings can be explained by the lipophilicity of the complexes (Table 1). The partition coefficient between water or buffer and n-octanol is the most widely used measure of chemical compound lipophilicity [24] since it is a major structural factor governing both pharmacokinetics and pharmacodynamics of drugs. The higher cytotoxicity for complex **(4)** can be associated with its higher log *P*, suggesting that the hydrophobicity increases its biological activity. The low cytotoxicity for complexes **(1–3)** could be explained by their low log *P*, compared to the lipophilicity of complex **(4)**.

### Cell migration and invasion

Metastatic process is described as a cascade of events where normal cells are transformed into tumor cells due to mutations in genes that regulate critical pathways, producing an imbalance between proliferation and cell death that eventually leads to the formation of a primary tumor. Further interactions with the stromal microenvironment surrounding tumor cells and extracellular matrix (ECM) proteins, contribute to the formation of new blood and lymphatic vessels. These interactions facilitate tumor cell migration and invasion of surrounding tissues, intravasation through newly formed vessels, and dissemination to other tissues, to form secondary tumors [25].

**Fig 3:**
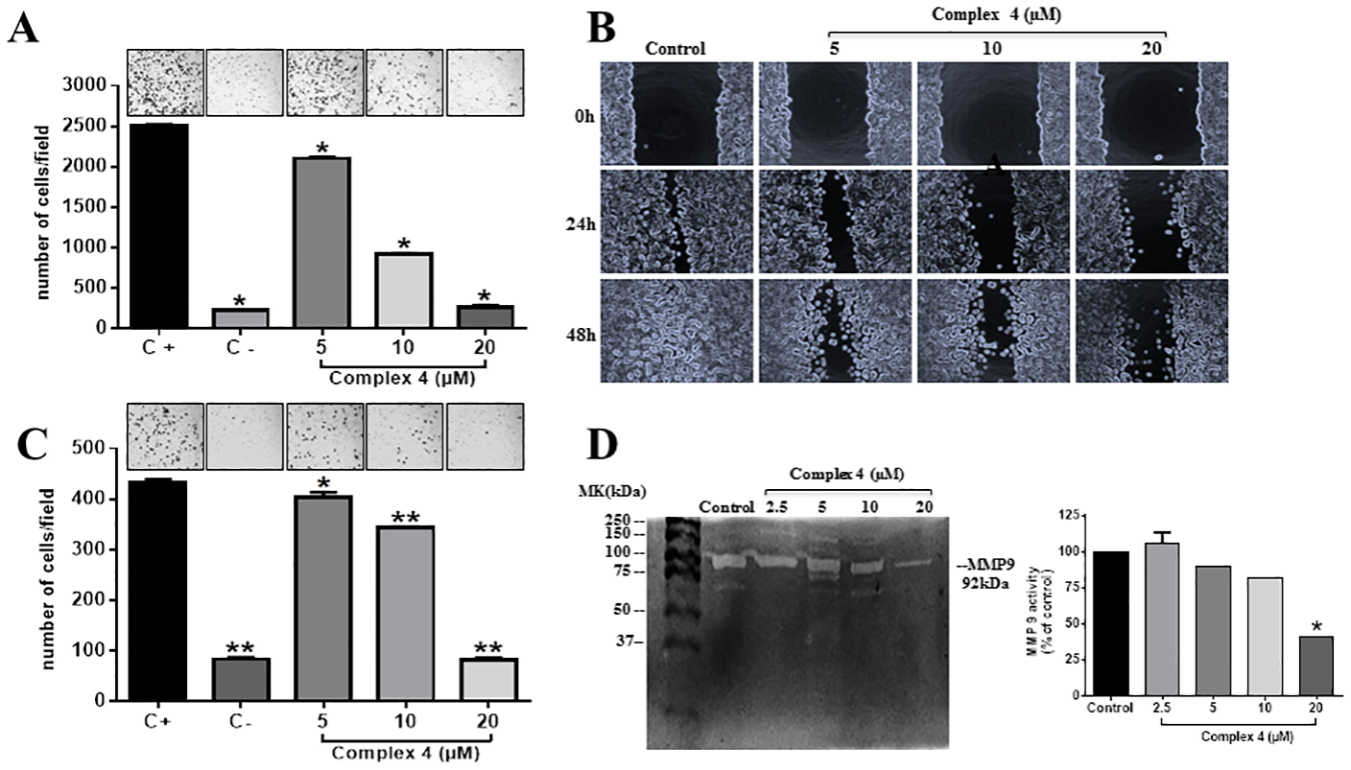
Effects of complex (4) on MDA-MB-231 cell migration and invasion. **(A)** Complex was incubated with MDA-MB-231 and cells were allowed to migrate in Boyden chambers. Migrated cells were fixed and quantified by manual counting. Representative images of the inserts are represented above each condition. The wells were photographed under a light microscope under 40× magnifications. Positive control (C+) represents migrating cells without any treatment and negative control (C-) was cells migrating toward an FBS-free medium. **(B)** Wound healing assay at 0, 24 and 48 h of treatment with the complex. **(C)** Effect of complex **(4)** on MDA-MB-231 cell invasion through matrigel. **(D)** Zymography in 1% gelatin-SDS-PAGE. A photograph of a representative zymography gel is shown. Gels were analyzed by densitometry, and data were normalized in percentage compared to untreated control cell lysate. Significant at the * *p*<0.005, **p<0.001 levels using ANOVA and Bonferroni tests.

The effects of complex **(4)** on MDA-MB-231 tumor cell migration were assessed using wound healing and Boyden chamber migration assays (Fig 3). Boyden Chamber and wound healing assays demonstrated that complex **(4)** inhibited MDA-MB-231 cell migration in a concentration dependent fashion (Fig 3A and 3B). One important characteristic of metastatic tumor is its ability to invade adjacent tissues. Compound **(4)** at concentration of 20 μM was able to inhibit the invasion MDA-MB-231 cells in approximately 80% compared to control (Fig 3C).

The invasion of tumor cells is mediated by matrix metalloproteinases (MMPs) such as MMP-2 and MMP-9. MMPs have a potential in ECM degradation and this correlates with the late stages of tumor invasion and metastasis [26]. The effects caused by the incubation of MDA-MB-231 cells with complex **(4)** on the activity of MMP-9 were accessed by zymography assay electrophoresis gel containing gelatin (Fig 3D). Complex **(4)** at concentrations 2.5, 5 and 10 μM did not inhibit the expression of MMP-9 in MDA-MB-231 cells. On the other hand, higher concentrations (20 μM) inhibited by 60% the expression of MMP-9 when compared to the untreated control (Fig 3D). It is important to point out that the effects of complex **(4)** were related to cell migration and invasion rather than to cell death, since all concentrations tested were not cytotoxic to the cells, as evaluated by cell morphology and proliferation assays (See Fig 2 and Table 1).

### Adhesion

**Fig 4:**
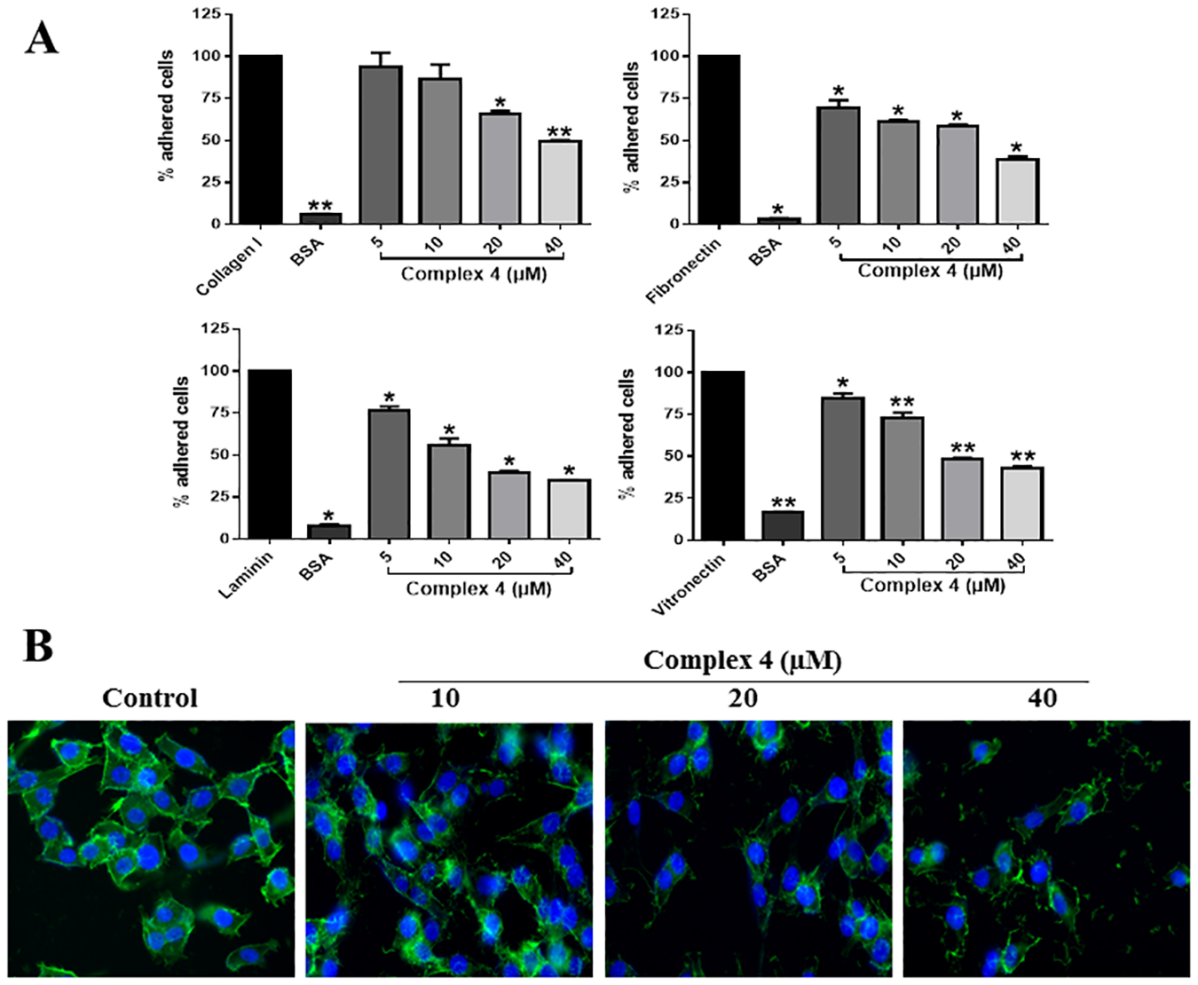
Effects of complex (4) on MDA-MB-231 cell adhesion and cytoskeleton structure. **(A)** Type I collagen, fibronectin, laminin and vitronectin were coated on the wells of a 96-well plate. Complex **(4)** was incubated with MDA-MB-231 cells, which were subsequently plated on the ECM coatings. After washes, remaining cells were quantified by colorimetry. **(B)** MDA-MB-231 cells were incubated with complex **(4)** and then with Phalloidin and DAPI. Significant at the *p<0.005, **p<0.001 levels using ANOVA and Bonferroni tests.

Cell adhesion plays a critical role in the metastatic process since there are molecular signals involved in cell-cell and cell-ECM adhesion that orchestrate tumor behaviours such as proliferation and invasion [27]. Cancer progression, response to therapy and metastasis depend on tumor microenvironment. Integrins are cell adhesion receptors that mediate interactions of cells with ECM [28]. The ability of the complex **(4)** to inhibit on MDA-MB-231 cell adhesion to type I collagen, fibronectin, laminin and vitronectin, was assessed by short-term adhesion assay (Fig 4A).

Complex **(4)** at concentrations 20 and 40 μM inhibited approximately 35% and 50% of MDA-MB-231 cell adhesion to type I collagen, respectively (Fig 4A). Complex **(4)** also inhibited cell adhesion to fibronectin, laminin and vitronectin at all concentrations tested. Higher concentrations (40 μM) inhibited about 65% of MDA-MB-231 cell adhesion to these ECM proteins, compared to control cells, without treatment (Fig 4A).

The mechanisms involved in this inhibition remain to be elucidated, however one hypothesis is that complex **(4)** could act by inhibiting the expression of adhesion molecules that mediate the binding of MDA-MB-231 to ECM proteins such as integrins or even bind to these integrins preventing cell adhesion. Whether the complex **(4)** binds to integrins needs to be further investigated. An additional hypothesis that may explain this effect is the potential effects of complex **(4)** on cytoskeleton proteins. The cytoskeleton provides the basic infrastructure for the maintenance of cell adhesion and motility. The cytoskeleton has various functions, such as organization of cytoplasmic organelles and intracellular compartments, segregation of chromosomes in mitosis events are responsible for determining the shape of the cell and promote cell-cell adhesion or cell-matrix by their interactions with cadherin and integrins, respectively [29].

To test this hypothesis, MDA-MB-231 cells and the cytoskeleton structure was analysed through phalloidin staining. The incubation of tumor cells with 10, 20 and 40 μM of complex **(4)** strongly modified the structure of the actin cytoskeleton, provoking a decrease in cytoskeleton density, at higher concentrations (20 and 40 μM) (Fig 4B). Such effect suggests that the inhibition of migration, invasion and adhesion by complex **(4)** may be related to its ability to modify the cytoskeleton structure. This result is corroborated by the work of Sava and colleagues, who demonstrated that NAMI-A ruthenium complex can modify the actin cytoskeleton structure, affect the function of integrins and adhesion of the HeLa cells [30].

### Apoptosis

**Fig 5:**
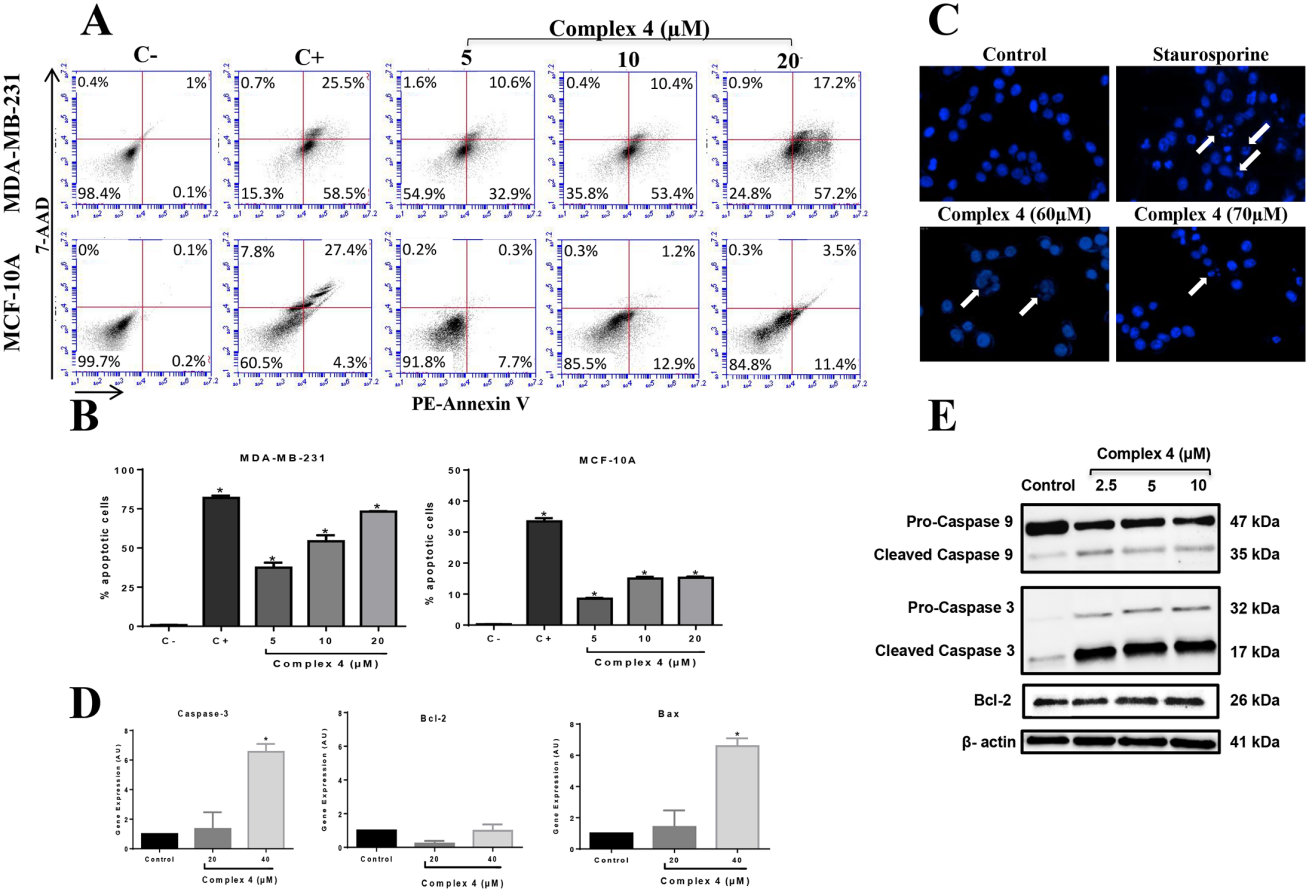
Effect of complex (4) on apoptosis in MDA-MB-231 breast tumor cells and MCF-10A non-tumor breast cells. **(A)** After treatment with the indicated concentrations of complex **(4)**, cells were incubated with PE-Annexin-V and 7AAD for 15min, harvested and then analyzed by cytometry. **(B)** The percentage of apoptotic and necrotic cells was plotted in a graph for MDA-MB-231 and MCF-10A cells. The fluorescence of 7AAD is detected in the FL3-A channel and the fluorescence of PE-Annexin-V is detected in the FL2-A channel. Camptothecin (campto) was used as a positive control for apoptosis. **(C)** Nuclear fragmentation promoted by complex **(4)** in MDA-MB-231 cells was investigated using DAPI staining. Staurosporine was used as a positive control for nuclear fragmentation. White arrows show fragmented nuclei. The expression of apoptotic and anti-apoptotic molecules was investigated by **(D)** qRT-PCR and **(E)** Western blotting analysis. Significant at the *p<0.005, **p<0.001, ***p<0.0001 levels using ANOVA and Bonferroni tests.

Evasion of apoptosis is one of the hallmarks of cancer and represents an important mechanism in clinical resistance to therapies [31]. Frequently, chemotherapy drives tumor cells to develop resistance to cytotoxic agents and radiation, consequently leading to resistance to apoptosis and in inefficiency in cancer treatment [32]. Ruthenium complexes that arrest cell cycle and/or (re)induce apoptosis in tumor cells could be a promising strategy for TNBC treatment. The apoptotic activity of complex **(4)** on the MDA-MB-231 and MCF-10A cells was analysed by flow cytometry using PE-Annexin-V kit Apoptosis Detection kit (BD Biosciences) (Fig 5A and 5B). Results show a concentration-dependent induction of MDA-MB-231 apoptosis, with 74.4% of tumor cells and only 14.9% of non-tumor cells suffering apoptosis after incubation with 20 μM of complex (4) (Fig 5A and 5B). These results suggest that malignant cells are more sensitive to complex **(4)** compared to non-tumor cells, for the induction of apoptosis. We also accessed the effects of complex **(4)** on apoptosis using DAPI staining (Fig 5C). The incubation of MDA-MB-231 cells with 60and 70 μM of complex **(4)** resulted in more apoptotic nucleic, compared to cells incubated control untreated cells. In agreement with our study, Nhukeaw and colleagues [33] evaluated the effects of metallo-intercalator ruthenium(II) complexes with the Clazpy ligand, [Ru(Clazpy)_**2**_bpy]Cl_**2**_.7H_**2**_O and [Ru(Clazpy)_**2**_phen]Cl_**2**_.8H_**2**_O in the induction of apoptosis in three breast tumor cell lines (MDA-MB-231, MCF-7 and HCC1932). A significant increase in MDA-MB-231 apoptotic cells was observed, with slightly less apoptotic rates in HCC1937 and MCF-7 cells, respectively.

We further investigated whether the apoptosis induction mediated by complex **(4)** would be related to apoptosis related gene expression investigating two pro-apoptotic genes (Bax and Caspase-3) and one anti-apoptotic gene (Bcl-2). Results indicate that complex **(4)** at concentration of 40 μM upregulated the expression of Bax and Caspase-3 genes, whereas did not affect the gene expression of Bcl-2 (Fig 5D). Western blotting analysis partially corroborates these results demonstrating that cleaved Caspase-3 and -9 protein levels are increased upon incubation with complex **(4)** even at low concentrations such as 2.5μM, with no in Bcl-2 protein levels (Fig 5E).

### Interaction studies with DNA and HSA

**Fig 6:**
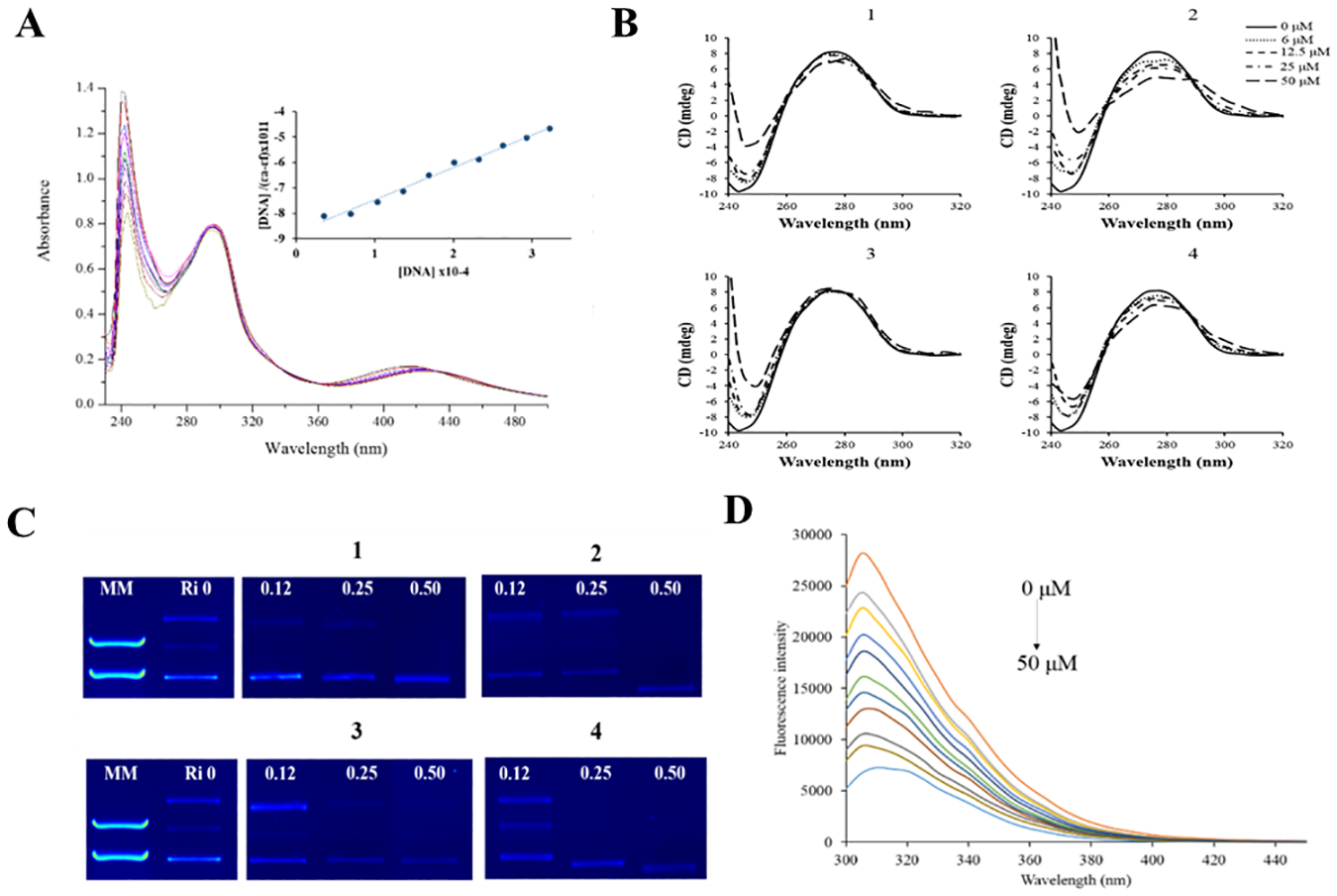
Absorption spectral titration. **(A)** Absorption spectral titration of complex **(4)** in the presence of increasing concentrations of *ct*-DNA at 298 K and fluorescence emission spectra of HSA at 37°C. Inset graph represents the plots of (ε_a_-ε_f_)/(ε_b_-ε_f_) versus [DNA] for the titration of DNA with Ru(II) complexes. **(B)** CD spectrum of *ct*-DNA (100 μM) in the presence of complexes **(1–4)** in Tris-HCl buffer after incubating by 18 h at 37°C. **(C)** Electrophoretic mobility pattern of pBR322 plasmid DNA incubated with metal complexes **(1–4)** by 18 h at 37°C, at indicated concentrations (μM). Ri = ratio complex/DNA; MM = molecular marker. **(D)** Fluorescence quenching spectra of HSA with different concentrations of complex (4) with the excitation wavelength at 270 nm at 37°C in a Trizma buffer, pH 7.4. The arrow shows the intensity changes upon increasing the concentration of the quencher (0 to 50 μM, orange line to light blue line, respectively).

The effects of complex **(4)** on tumor cell apoptosis presuppose their ability to bind to DNA. Ru(II) complexes can bind DNA in covalent or non-covalent interactions fashion such as grooving binding, electrostatic binding and intercalation [34]. To explore the possibility of DNA as a potential target for the complexes, spectroscopic studies, circular dichroism and gel electrophoresis were carried out (Fig 6). In 10% DMSO and 5 mM Tris-HCl, 50mM NaCl buffer at pH 7.4 the complexes **(1–4)** display a higher energy band in 240 nm, which arises from intra-ligand π–π* type transitions involving ligand energy levels, the MLCT bands appear in the region of the spectrum typical for Ru(II) complexes with coordinated phosphine ligands. Upon the addition of calf thymus DNA (*ct*-DNA) interesting changes in intensity of these bands are observed. All complexes exhibit hypochromism at increasing *ct-*DNA concentrations, and only for complex **(4)** isosbestic points at 338, 358 and 435 nm (Fig 6A). The equilibrium constant for binding of **(1–4)** to *ct*-DNA was evaluated by monitoring the decrease in absorbance at 240 nm and fitting these data to neighbor exclusion equation. The values obtained here (0.98–1.34x10^**3**^ M^**-1**^) are lower than that reported for classical intercalators (for ethidium bromide and [Ru(phen)_**2**_(dppz)]^**2+**^) whose binding constants have been found to be in the order of 10^**6**^ to 10^**7**^ M^**-1**^.

The CD spectral technique is very sensitive for diagnosing changes in the secondary structure of DNA, resulting from drug-DNA interactions. A typical CD spectrum of *ct*-DNA shows a maximum at 275 nm, due to the base stacking and a minimum at 248 nm attributed to the right-handed helicity, characteristic of the B conformation. To determine whether the Ru(II) complexes studied here cause changes in the helical structure of DNA, CD spectra of *ct*-DNA with increasing concentrations of compounds **(1–4)** were acquired, up to molar ratio drug/DNA (Ri): 0.06 to 0.50. A small decrease in the CD magnitude of positive band was observed, a higher distortion of the negative band can be related with contribution to the absorption of the complexes (Fig 6B).

The influence of the compounds on the tertiary structure of DNA was determined by their ability to modify the electrophoretic mobility of forms of pBR322 plasmid DNA (Fig 6C). Agarose gel electrophoresis was performed using pBR322 plasmid DNA in the absence and presence of the complexes. Thus simple groove binding and electrostatic interaction of small molecules shows less or no perturbation on the base-stacking and helicity bands [35]. The electrophorogram shows that the increase in the concentration of each complex caused changes in the mobility of the plasmid. In Ri 0.12 and 0.25 complexes **(1–3)** show the corresponding open circular, linear and super coiled form. In contrast, complex **(4)** only shows these bands in Ri 0.12; there is also a decrease in intensity of both bands. More interestingly, when the Ri is 0.50, complexes only show the super coiled form and it is observed also that complexes **(2)** and **(4)** increased the electrophoretic mobility of this band (Fig 6C). The behavior observed for the electrophoretic mobility for the complexes **(1–4)** indicates that some conformational changes occurred. This means that the degree of super helicity of the DNA molecules has been altered, in the same way as that described previously for other coordination platinum(II) and palladium (II) compounds containing 7-azaindolyl ligand [36].

Human serum albumin (HSA) is the most abundant protein in the blood and cerebrospinal fluid and plays a fundamental role in the distribution of essential transition metal ions in the human body. Moreover, HSA is an important physiological transporter of the essential metal ions Cu^**2+**^, and Zn^**2+**^ in the bloodstream. In addition, HSA is one of the main targets and hence most studied binding protein for metallodrugs based on complexes with Au, Pt, Ru and V [37]. Fluorimetric procedures, which are simple and sensitive, have been developed for the quantitation of a wide variety of substances of biological importance, including HSA that exhibit fluorescence in the ultraviolet region. The fluorescence spectrum is essentially that of tryptophan residue; contribution of tyrosine is relatively minor. In order to determine the protein binding affinity of complexes **(1–4)**, tryptophan emission quenching experiments were carried out by adding the complexes in increasing concentration (0–50 μM) to HSA at two different temperatures and following the decrease in fluorescence intensities (Fig 6D). The results point out a good linearity at the two different temperatures. The binding constants, K_**SV**_, calculated from the Stern-Volmer equation are presented on Table 2. The results show that the K_**SV**_ for **(2–3)** is correlated with temperature, which indicates that this quenching mechanism is probably due to a dynamic collision.

**Table 2:** Interactions with DNA and HSA. Binding constants for the interaction between Ru(II) complexes **(1–4)** and calf thymus *ct*-DNA and stern-Volmer quenching constant (K_sv_, M^-1^), biomolecular quenching rate constant (Kq, M^-1^s^-1^), binding constant (Kb, M^-1^), the number of binding sites (n), ΔG(KJ.mol^-1^), ΔH (KJ.mol^-1^) and ΔS (J.mol^-1^K) values for the complex-HSA system at different temperatures.

Complex	DNA			HSA						
	Kb (x10^3^)	% H	T	K_sv_ (x10^4^)	Kq (x10^12^)	Kb (x10^5^)	n	ΔG	ΔH	ΔS
1	1.34 ± 0.02	51.45 ± 2.15	298	5.04 ± 0.09	52.60	0.90 ± 0.05	1.10	- 28.50	6.10	72.70
			310	4.45 ± 0.18	46.50	0.65 ± 0.03	1.06	- 28.60		
2	0.98 ± 0.01	53.04 ± 0.20	298	2.81 ± 0.12	29.35	0.30 ± 0.01	1.00	- 25.53	1.17	85.80
			310	3.28 ± 0.14	34.25	0.25 ± 0.02	0.98	- 26.55		
3	1.07 ± 0.03	58.55 ± 0.92	298	1.56 ± 0.13	16.30	0.05 ± 0.003	0.90	- 21.13	4.25	80.00
			310	1.63 ± 0.10	17.02	0.03 ± 0.003	0.85	- 20.50		
4	1.30 ± 0.02	37.80 ± 1.00	298	4.74 ± 0.70	49.50	3.84 ± 0.05	1.20	- 31.85	1.50	110.13

In contrast, in the interaction of **(1–4)** with HSA the K_SV_ reveals an inverse correlation with temperature, which indicates that probably, this quenching mechanism is static, initiated by an adduct formation. Moreover, the values of K_q_ were in the range of (16.3–52.6 x 10^12^ M^-1^s^-1^) for all complexes, which is far higher than 2.0×10^10^ M^-1^s^-1^, the maximum possible value for dynamic quenching, indicating the existence of a static quenching mechanism. The number of binding sites between HSA and **(1–4)** complexes is approximately equal to 1, indicating that there is only one binding site in the HSA for each complex. The thermodynamic parameters ΔH, ΔS and ΔG are the main evidence for confirming the binding modes. From the thermodynamic standpoint, ΔH > 0 and ΔS > 0 implies a hydrophobic interaction; ΔH < 0 and ΔS < 0 reflects the van der Waals force or hydrogen bond formation; and ΔH < 0 and ΔS > 0 suggests an electrostatic force.

As observed in Table 2, the positive ΔH and ΔS values reveal the predominance of hydrophobic interactions of the compounds with HSA. Furthermore, the negative ΔG values demonstrate that the interaction process is spontaneous. The magnitude of the HSA-binding constant of complexes **(1–4)** compared to other metals complexes suggests a moderate interaction with HSA molecule [38].

Overall, there are little studies comparing the effects of Ru complexes on tumor and non-tumor cell lines (for a review, see [39]). In general, in the present study we demonstrated that complex **(4)** was able to inhibit cell migration and invasion of TNBC cells, at concentrations as low as 5μM. In addition, complex **(4)** inhibited TNBC cell adhesion to different ECM components and this effect was mediated, at least partially, due to its interference with actin cytoskeleton. Other Ru complexes were able to interfere with cell migration, invasion and adhesion processes in tumor cells, at concentrations ranging from 0.75μM to 4μM, depending on the assay [39]. Complex **(4)** induced apoptosis in TNBC cells, as demonstrated in our study by flow cytometry, DAPI staining, RT-qPCR and western blotting analyses at concentrations from 2.5μM. Several other studies demonstrated that Ru complexes induce apoptosis on TNBC cells, through different mechanisms. To give few examples, co-treatment cells with RuPOP and TRAIL effectively triggered apoptosis through the activation of caspases-3/-8/-9 and cleaved PARP in MDA-MB-231 cells at concentrations as low as 2μM [40]. In contrast, Zeng and co-workers [41] found that 40μM of Λ-RM0627 exhibited little apoptosis inducing effect on MDA-MB-231 cells, with only 6.2% cells in the late stage and 21.8% cells in the early stage of apoptosis.

## Conclusions

Taken together, the results show that complex **(4)** is more lipophilic and cytotoxic than complexes **(1–3).** Complex **(4)** is able to inhibit MDA-MB-231 cell proliferation, adhesion, migration and invasion. Furthermore, complex **(4)** modifies the structure of the actin cytoskeleton, binds to DNA, inducing apoptosis and inhibits MMP-9 secretion in this cell line. This work supports the evidence that complex **(4)** should be further studied in order to explore its potential of action using *in vivo* models, which may contribute to the development of a new antitumor drug to be applied in chemotherapy.

## Supporting information

S1 Table Selected bond lengths [Å] and angles [°] of the complexes (1) [Ru(SO_4_)(dppb)(bipy)], (2) [Ru(CO_3_)(dppb)(bipy)], (3) [Ru(C_2_O_4_)(dppb)(bipy)] and (4) [Ru(CH_3_COO)(dppb)(bipy)]PF_6_. (DOCX)

S2 Table Crystal data and refinement parameters of the complexes (1–4). (DOCX)

S1 Fig Synthetic route used to obtain the complexes [Ru(SO_4_)(dppb)(bipy)] (1), [Ru(CO_3_)(dppb)(bipy)] (2), [Ru(C_2_O_4_)(dppb)(bipy)] (3) and [Ru(CH_3_CO_2_)(dppb)(bipy)]PF_6_ (4), from the precursor [RuCl_2_(dppb)(bipy)]. (TIF)

S2 Fig Cellular morphology of MDA-MB-231, MCF-7 and MCF-10A cells, treated with complex (1). (TIF)

S3 Fig Cellular morphology of MDA-MB-231, MCF-7 and MCF-10A cells, treated with complex (2). (TIF)

S4 Fig Cellular morphology of MDA-MB-231, MCF-7 and MCF-10A cells, treated with complex (3). (TIF)

## References

[1] Ban KA, Godellas CV. Epidemiology of breast cancer. Surgical oncology clinics of North America. 2014;23(3):409–22. doi: 10.1016/j.soc.2014.03.011 . 24882341 10.1016/j.soc.2014.03.011

[2] Vidal SJ, Rodriguez-Bravo V, Galsky M, Cordon-Cardo C, Domingo-Domenech J. Targeting cancer stem cells to suppress acquired chemotherapy resistance. Oncogene. 2014;33(36):4451–63. doi: 10.1038/onc.2013.411 . 24096485 10.1038/onc.2013.411

[3] Norum JH, Andersen K, Sorlie T. Lessons learned from the intrinsic subtypes of breast cancer in the quest for precision therapy. The British journal of surgery. 2014;101(8):925–38. doi: 10.1002/bjs.9562 . 24849143 10.1002/bjs.9562

[4] Hongthong K, Ratanaphan A. BRCA1-Associated Triple-Negative Breast Cancer and Potential Treatment for Ruthenium-Based Compounds. Current cancer drug targets. 2016;16(7):606–17. . 26845433 10.2174/1568009616666160203113957

[5] Abramson VG, Lehmann BD, Ballinger TJ, Pietenpol JA. Subtyping of triple-negative breast cancer: implications for therapy. Cancer. 2015;121(1):8–16. doi: 10.1002/cncr.28914 ; 25043972 10.1002/cncr.28914 PMC4270831

[6] Ciarimboli G. Membrane transporters as mediators of cisplatin side-effects. Anticancer research. 2014;34(1):547–50. . 24403515

[7] Leijen S, Burgers SA, Baas P, Pluim D, Tibben M, van Werkhoven E, et al Phase I/II study with ruthenium compound NAMI-A and gemcitabine in patients with non-small cell lung cancer after first line therapy. Investigational new drugs. 2015;33(1):201–14. doi: 10.1007/s10637-014-0179-1 . 25344453 10.1007/s10637-014-0179-1

[8] Hartinger CG, Zorbas-Seifried S, Jakupec MA, Kynast B, Zorbas H, Keppler BK. From bench to bedside—preclinical and early clinical development of the anticancer agent indazolium trans-[tetrachlorobis(1H-indazole)ruthenate(III)] (KP1019 or FFC14A). Journal of inorganic biochemistry. 2006;100(5–6):891–904. doi: 10.1016/j.jinorgbio.2006.02.013 . 16603249 10.1016/j.jinorgbio.2006.02.013

[9] Wu Q, He J, Mei W, Zhang Z, Wu X, Sun F. Arene ruthenium(ii) complex, a potent inhibitor against proliferation, migration and invasion of breast cancer cells, reduces stress fibers, focal adhesions and invadopodia. Metallomics: integrated biometal science. 2014;6(12):2204–12. doi: 10.1039/c4mt00158c . 25142071 10.1039/c4mt00158c

[10] Barbosa MI, Correa RS, de Oliveira KM, Rodrigues C, Ellena J, Nascimento OR, et al Antiparasitic activities of novel ruthenium/lapachol complexes. Journal of inorganic biochemistry. 2014;136:33–9. doi: 10.1016/j.jinorgbio.2014.03.009 . 24727183 10.1016/j.jinorgbio.2014.03.009

[11] Almada da Silva J, Becceneri AB, Sanches Mutti H, Moreno Martin AC, Fernandes da Silva MF, Fernandes JB, et al Purification and differential biological effects of ginger-derived substances on normal and tumor cell lines. J Chromatogr B Analyt Technol Biomed Life Sci. 2012;903:157–62. doi: 10.1016/j.jchromb.2012.07.013 . 22858304 10.1016/j.jchromb.2012.07.013

[12] Yue PY, Leung EP, Mak NK, Wong RN. A simplified method for quantifying cell migration/wound healing in 96-well plates. Journal of biomolecular screening. 2010;15(4):427–33. doi: 10.1177/1087057110361772 . 20208035 10.1177/1087057110361772

[13] Selistre-de-Araujo HS, Cominetti MR, Terruggi CH, Mariano-Oliveira A, De Freitas MS, Crepin M, et al Alternagin-C, a disintegrin-like protein from the venom of Bothrops alternatus, modulates alpha2beta1 integrin-mediated cell adhesion, migration and proliferation. Brazilian journal of medical and biological research = Revista brasileira de pesquisas medicas e biologicas / Sociedade Brasileira de Biofisica [et al]. 2005;38(10):1505–11. doi: /S0100-879X2005001000007 . 16172743 10.1590/s0100-879x2005001000007

[14] Fuzer AM, Filho JC, Becceneri AB, Dos Santos DA, da Silva MF, Vieira PC, et al Effects of limonoid cedrelone on MDA-MB-231 breast tumor cells in vitro. Anti-cancer agents in medicinal chemistry. 2013;13(10):1645–53. . 23869780 10.2174/18715206113139990314

[15] Leber TM, Negus RP. Detection and quantitation of matrix metalloproteases by zymography. Methods in molecular medicine. 2001;39:509–14. doi: 10.1385/1-59259-071-3:509 . 21340807 10.1385/1-59259-071-3:509

[16] Cominetti MR, Ribeiro JU, Fox JW, Selistre-de-Araujo HS. BaG, a new dimeric metalloproteinase/disintegrin from the Bothrops alternatus snake venom that interacts with alpha5beta1 integrin. Archives of biochemistry and biophysics. 2003;416(2):171–9. . 12893294 10.1016/s0003-9861(03)00298-4

[17] Livak KJ, Schmittgen TD. Analysis of relative gene expression data using real-time quantitative PCR and the 2(-Delta Delta C(T)) Method. Methods. 2001;25(4):402–8. doi: 10.1006/meth.2001.1262 . 11846609 10.1006/meth.2001.1262

[18] Bustin SA, Benes V, Garson JA, Hellemans J, Huggett J, Kubista M, et al The MIQE guidelines: minimum information for publication of quantitative real-time PCR experiments. Clinical chemistry. 2009;55(4):611–22. doi: 10.1373/clinchem.2008.112797 . 19246619 10.1373/clinchem.2008.112797

[19] Baka E, Comer JE, Takacs-Novak K. Study of equilibrium solubility measurement by saturation shake-flask method using hydrochlorothiazide as model compound. Journal of pharmaceutical and biomedical analysis. 2008;46(2):335–41. doi: 10.1016/j.jpba.2007.10.030 . 18055153 10.1016/j.jpba.2007.10.030

[20] Queiroz SL, Batista AA, Oliva G, Gambardella MT, Santos RHA, MacFarlane KS, et al The reactivity of five-coordinate Ru(II) (1,4-bis(diphenylphosphino)butane) complexes with the N-donor ligands: ammonia, pyridine, 4-substituted pyridines, 2,2′-bipyridine, bis(o-pyridyl)amine, 1,10-phenanthroline, 4,7-diphenylphenanthroline and ethylenediamine. Inorganica Chimica Acta. 1998;267(2):209–21.

[21] Correa RS, da Silva MM, Graminha AE, Meira CS, Santos JA, Moreira DR, et al Ruthenium(II) complexes of 1,3-thiazolidine-2-thione: Cytotoxicity against tumor cells and anti-Trypanosoma cruzi activity enhanced upon combination with benznidazole. J Inorg Biochem. 2016;156:153–63. doi: 10.1016/j.jinorgbio.2015.12.024 . 26795676 10.1016/j.jinorgbio.2015.12.024

[22] Buss I, Garmann D, Galanski M, Weber G, Kalayda GV, Keppler BK, et al Enhancing lipophilicity as a strategy to overcome resistance against platinum complexes? Journal of inorganic biochemistry. 2011;105(5):709–17. doi: 10.1016/j.jinorgbio.2011.02.005 . 21450275 10.1016/j.jinorgbio.2011.02.005

[23] Alberts DS, Samon SE, Chen HS, Surwit EA, Soehnlen B, Young L, et al In-vitro clonogenic assay for predicting response of ovarian cancer to chemotherapy. Lancet. 1980;2(8190):340–2. . 6105478 10.1016/s0140-6736(80)90340-2

[24] McKeage MJ, Berners-Price SJ, Galettis P, Bowen RJ, Brouwer W, Ding L, et al Role of lipophilicity in determining cellular uptake and antitumour activity of gold phosphine complexes. Cancer chemotherapy and pharmacology. 2000;46(5):343–50. doi: 10.1007/s002800000166 . 11127937 10.1007/s002800000166

[25] Quail DF, Joyce JA. Microenvironmental regulation of tumor progression and metastasis. Nature medicine. 2013;19(11):1423–37. doi: 10.1038/nm.3394 ; 24202395 10.1038/nm.3394 PMC3954707

[26] Brown GT, Murray GI. Current mechanistic insights into the roles of matrix metalloproteinases in tumour invasion and metastasis. The Journal of pathology. 2015;237(3):273–81. doi: 10.1002/path.4586 . 26174849 10.1002/path.4586

[27] Ellis SJ, Tanentzapf G. Integrin-mediated adhesion and stem-cell-niche interactions. Cell and tissue research. 2010;339(1):121–30. doi: 10.1007/s00441-009-0828-4 . 19588168 10.1007/s00441-009-0828-4

[28] Gehler S, Ponik SM, Riching KM, Keely PJ. Bi-directional signaling: extracellular matrix and integrin regulation of breast tumor progression. Critical reviews in eukaryotic gene expression. 2013;23(2):139–57. . 23582036 10.1615/critreveukargeneexpr.2013006647 PMC5055378

[29] Hall A. The cytoskeleton and cancer. Cancer metastasis reviews. 2009;28(1–2):5–14. doi: 10.1007/s10555-008-9166-3 . 19153674 10.1007/s10555-008-9166-3

[30] Sava G, Frausin F, Cocchietto M, Vita F, Podda E, Spessotto P, et al Actin-dependent tumour cell adhesion after short-term exposure to the antimetastasis ruthenium complex NAMI-A. European journal of cancer. 2004;40(9):1383–96. doi: 10.1016/j.ejca.2004.01.034 . 15177498 10.1016/j.ejca.2004.01.034

[31] MacFarlane M, Williams AC. Apoptosis and disease: a life or death decision. EMBO reports. 2004;5(7):674–8. doi: 10.1038/sj.embor.7400191 ; 15218528 10.1038/sj.embor.7400191 PMC1299101

[32] Fesik SW. Promoting apoptosis as a strategy for cancer drug discovery. Nature reviews Cancer. 2005;5(11):876–85. doi: 10.1038/nrc1736 . 16239906 10.1038/nrc1736

[33] Nhukeaw T, Temboot P, Hansongnern K, Ratanaphan A. Cellular responses of BRCA1-defective and triple-negative breast cancer cells and in vitro BRCA1 interactions induced by metallo-intercalator ruthenium(II) complexes containing chloro-substituted phenylazopyridine. BMC cancer. 2014;14:73 doi: 10.1186/1471-2407-14-73 ; 24507701 10.1186/1471-2407-14-73 PMC3933379

[34] Brabec V, Novakova O. DNA binding mode of ruthenium complexes and relationship to tumor cell toxicity. Drug resistance updates: reviews and commentaries in antimicrobial and anticancer chemotherapy. 2006;9(3):111–22. doi: 10.1016/j.drup.2006.05.002 . 16790363 10.1016/j.drup.2006.05.002

[35] Ratanaphan A, Nhukeaw T, Hongthong K, Dyson PJ. Differential Cytotoxicity, Cellular Uptake, Apoptosis and Inhibition of BRCA1 Expression of BRCA1-Defective and Sporadic Breast Cancer Cells Induced by an Anticancer Ruthenium(II)-Arene Compound, RAPTA-EA1. Anti-cancer agents in medicinal chemistry. 2016 . 27039925 10.2174/1871520616666160404110953

[36] Pages BJ, Ang DL, Wright EP, Aldrich-Wright JR. Metal complex interactions with DNA. Dalton transactions. 2015;44(8):3505–26. doi: 10.1039/c4dt02700k . 25427534 10.1039/c4dt02700k

[37] Gou Y, Zhang Y, Yang F, Liang H. Evaluation of interactions between platinum-/ruthenium-based anticancer agents and human serum albumin: development of HSA carrier for metal-based drugs. Current pharmaceutical design. 2015;21(14):1848–61. . 25732554 10.2174/1381612821666150302114739

[38] Carreira M, Calvo-Sanjuan R, Sanau M, Zhao X, Magliozzo RS, Marzo I, et al Cytotoxic hydrophilic iminophosphorane coordination compounds of d(8) metals. Studies of their interactions with DNA and HSA. Journal of inorganic biochemistry. 2012;116:204–14. doi: 10.1016/j.jinorgbio.2012.06.017 ; 23063789 10.1016/j.jinorgbio.2012.06.017 PMC3483362

[39] Popolin CP, Cominetti MR. A review of ruthenium complexes activities on different steps of the metastatic process in breast cancer cells. Mini Rev Med Chem. 2017 doi: 10.2174/1389557517666170206151218 . 28176627 10.2174/1389557517666170206151218

[40] Cao W, Zheng W, Chen T. Ruthenium polypyridyl complex inhibits growth and metastasis of breast cancer cells by suppressing FAK signaling with enhancement of TRAIL-induced apoptosis. Sci Rep. 2015;5:9157 doi: 10.1038/srep09157 ; 25778692 10.1038/srep09157 PMC4361883

[41] Zeng ZP, Wu Q, Sun FY, Zheng KD, Mei WJ. Imaging Nuclei of MDA-MB-231 Breast Cancer Cells by Chiral Ruthenium(II) Complex Coordinated by 2-(4-Phenyacetylenephenyl)-1H-imidazo[4,5f][1,10]phenanthroline. Inorg Chem. 2016;55(11):5710–8. doi: 10.1021/acs.inorgchem.6b00824 . 27191197 10.1021/acs.inorgchem.6b00824

