# Supplementary material for: Cytotoxicity and anti-tumor effects of new ruthenium complexes on triple negative breast cancer cells
Source: PLoS One. 2017 Sep 12;12(9):e0183275. doi: 10.1371/journal.pone.0183275 (PMC5595280; doi:10.1371/journal.pone.0183275)
Supplement: S1 Table — (DOCX) [file pone.0183275.s001.docx]

|  | **Complexes** | | | |
| --- | --- | --- | --- | --- |
| **Fragment** | **1** | **2** | **3** | **4** |
| **Ru-O(1)** | 2.163(6) | 2.1519(19) | 2.116(3) | 2.2245(18) |
| **Ru-O(2)** | 2.150(5) | 2.090(2) | 2.104(3) | 2.1210(17) |
| **Ru-N(1)** | 2.044(6) | 2.084(2) | 2.078(4) | 2.061(2) |
| **Ru-N(2)** | 2.089(8) | 2.104(2) | 2.093(4) | 2.092(2) |
| **Ru-P(1)** | 2.285(2) | 2.2671(8) | 2.2872(12) | 2.3212(7) |
| **Ru-P(2)** | 2.334(2) | 2.3053(8) | 2.3064(12) | 2.2732(7) |
| **S(1)-O(1)** | 1.527(6) | - | - | - |
| **S(1)-O(2)** | 1.529(6) | - | - | - |
| **S(1)-O(3)** | 1.458(7) | - | - | - |
| **S(1)-O(4)** | 1.434(7) | - | - | - |
| **C(1)-O(1)** | - | 1.325(4) | 1.273(6) | - |
| **C(1)-O(3)** | - | 1.246(4) | 1.227(6) | - |
| **C(1)-O(2)** | - | 1.302(4) | - | - |
| **O(2)-C(2)** | - | - | 1.290(6) | - |
| **O(4)-C(2)** | - | - | 1.220(6) | - |
| **C(11)-O(1)** | - | - | - | 1.277(3) |
| **C(11)-O(2)** | - | - | - | 1.277(3) |
|  |  | **Bond angles** |  |  |
| **N(1)-Ru-N(2)** | 78.3(3) | 77.83(11) | 78.30(16) | 79.26(8) |
| **N(1)-Ru-O(2)** | 97.4(2) | 160.73(9) | 163.04(14) | 163.34(8) |
| **N(2)-Ru-O(2)** | 83.5(2) | 88.58(9) | 88.14(15) | 89.47(7) |
| **N(1)-Ru-O(1)** | 160.1(2) | 102.57(9) | 89.91(15) | 105.36(8) |
| **N(2)-Ru-O(1)** | 88.1(3) | 84.81(8) | 83.76(14) | 82.37(8) |
| **O(2)-Ru-O(1)** | 66.3(2) | 62.16(8) | 78.42(13) | 60.56(7) |
| **N(1)-Ru-P(2)** | 97.4(2) | 103.65(8) | 104.36(11) | 87.44(6) |
| **N(2)-Ru-P(2)** | 170.9(2) | 173.81(7) | 171.86(11) | 91.71(6) |
| **O(2)-Ru-P(2)** | 89.16(17) | 88.49(6) | 87.76(10) | 105.28(5) |
| **O(1)-Ru-P(2)** | 93.86(17) | 89.01(6) | 88.53(10) | 164.50(5) |
| **N(1)-Ru-P(1)** | 87.66(19) | 87.59(7) | 87.66(12) | 99.50(6) |
