# Supplementary material for: Cytotoxicity and anti-tumor effects of new ruthenium complexes on triple negative breast cancer cells
Source: PLoS One. 2017 Sep 12;12(9):e0183275. doi: 10.1371/journal.pone.0183275 (PMC5595280; doi:10.1371/journal.pone.0183275)
Supplement: S2 Table — (DOCX) [file pone.0183275.s002.docx]

| **Compound** | **(1)** | **(2)** | **(3)** | **(4)** |
| --- | --- | --- | --- | --- |
| **Empirical formula** | C_38_H_36_N_2_O_4_P_2_SRu | C_39_H_44_N_2_O_7_P_2_Ru | C_40_H_40_N_2_O_6_P_2_Ru | C_40_H_39_N_2_O_2_F_6_P_3_Ru |
| **Temperature (K)** | 293(2) | 293(2) | 293(2) | 100(2) |
| **FW** | 779.795 | 815.77 | 807.75 | 887.71 |
| **Crystal system** | Orthorhombic | Triclinic | Monoclinic | Monoclinic |
| **Space group** | *Pca2_1_* | *P-1* | *P2_1_* | *P2_1_/n* |
| **a (Å)** | 17.4128(5) | 9.7367(6) | 13.3670(5) | 11.5648(1) |
| **b (Å)** | 17.5802(8) | 13.0458(9) | 9.8760(4) | 19.9168(3) |
| **c (Å)** | 24.4783(8) | 15.8811(15) | 14.5480(6) | 17.0575(3) |
| **α (°)** | 90 | 80.580(4) | 90 | 90 |
| **β (°)** | 90 | 87.855(3) | 103.265(2) | 102.149(1) |
| **γ(°)** | 90 | 68.335(3) | 90 | 90 |
| **V (Å^3^)** | 7493.3(5) | 1848.9(2) | 1869.28(13) | 3840.92(9) |
| **Z** | 4 | 2 | 2 | 4 |
| **D. calc (mg/m^3^)** | 1.420 | 1.465 | 1.435 | 1.535 |
| **Absorption coefficient (mm^−1^)** | 0.603 | 0.563 | 0.554 | 0.601 |
| **F(000)** | 3296 | 844 | 832 | 1808 |
| **θ range for data collection (°)** | 2.991 to 25.232 | 3.283 to 26.090 | 2.943 to 26.894 | 3.19 to 26.63 |
| **Index ranges** | -20<=h<=20 | -12≤h≤12 | -17≤h≤17 | -14 ≤ *h* ≤ 14 |
|  | -20<=k<=17 | -16≤k≤16 | -12≤k≤12 | -21 ≤ *k* ≤ 25 |
|  | -29<=l<=27 | -19≤l≤18 | -18≤l≤18 | -21 ≤ *l* ≤ 21 |
| **Reflections collected** | 33094 | 13379 | 23406 | 26398 |
| **Independent reflections (Rint)** | 12304 [R(int) = 0.0685] | 7311 [R(int) = 0.0407] | 7728 [R(int) = 0.0771] | 8044[R(int) = 0.0565] |
| **Goodness-of-fit on F^2^** | 0.981 | 0.996 | 1.018 | 1.039 |
| **Final R index**  **[I>2(I)]** | R1 = 0.0488  wR2 = 0.0993 | R1 = 0.0399  wR2 = 0.0830 | R1 = 0.0373  wR2 = 0.0925 | R1 = 0.0382  wR2 = 0.0878 |
| **R indices (all data)** | R1 = 0.0784  wR2 = 0.1076 | R1 = 0.0639  wR2 = 0.0893 | R1 = 0.0445  wR2 = 0.0963 | R1 = 0.0610  wR2 = 0.0966 |
