# Supplementary figures and images for: Cytotoxicity and anti-tumor effects of new ruthenium complexes on triple negative breast cancer cells
Source: PLoS One. 2017 Sep 12;12(9):e0183275. doi: 10.1371/journal.pone.0183275 (PMC5595280; doi:10.1371/journal.pone.0183275)

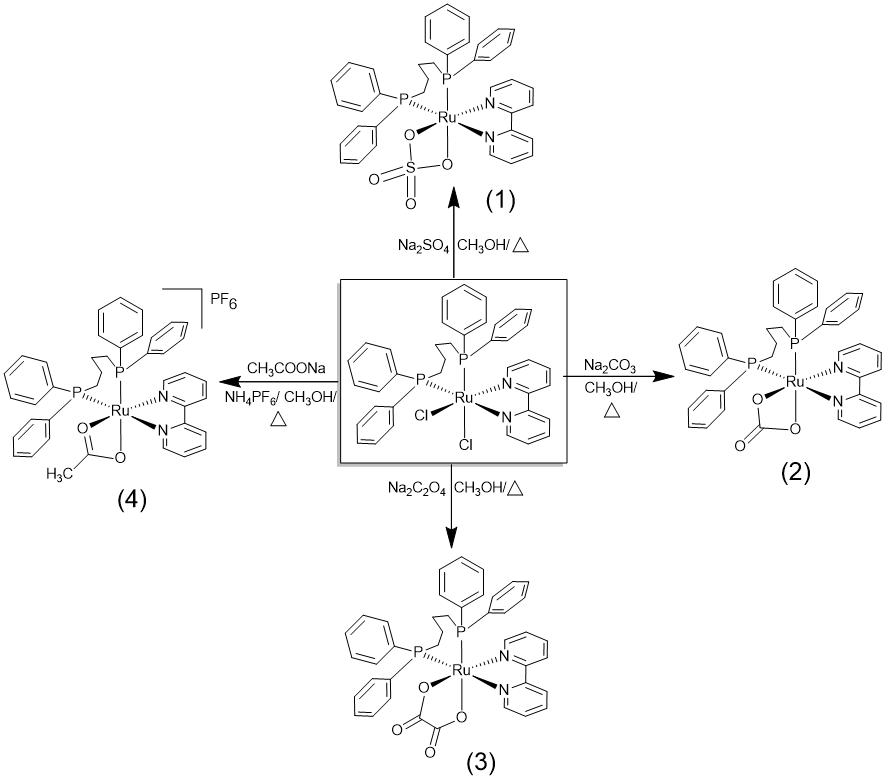

Supplement: S1 Fig — (TIF) [file pone.0183275.s003.tif]

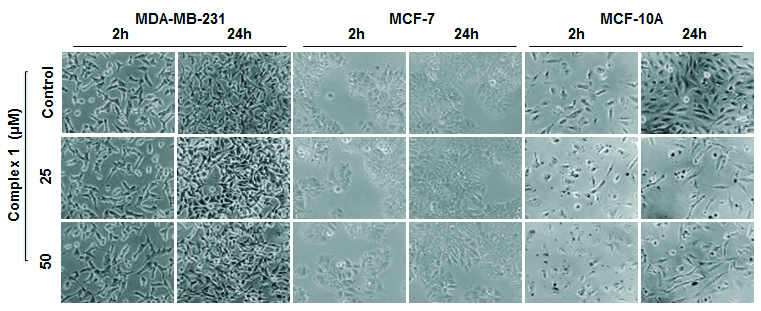

Supplement: S2 Fig — (TIF) [file pone.0183275.s004.tif]

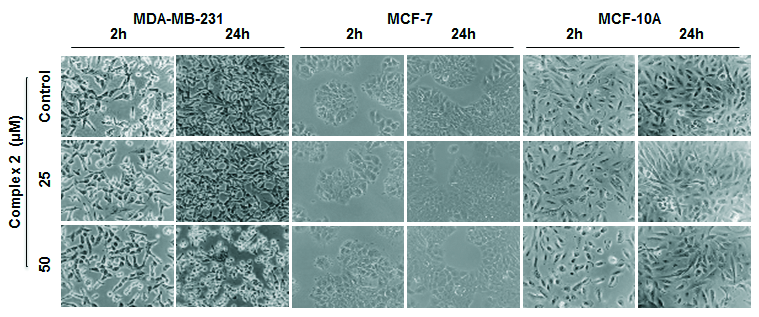

Supplement: S3 Fig — (TIF) [file pone.0183275.s005.tif]

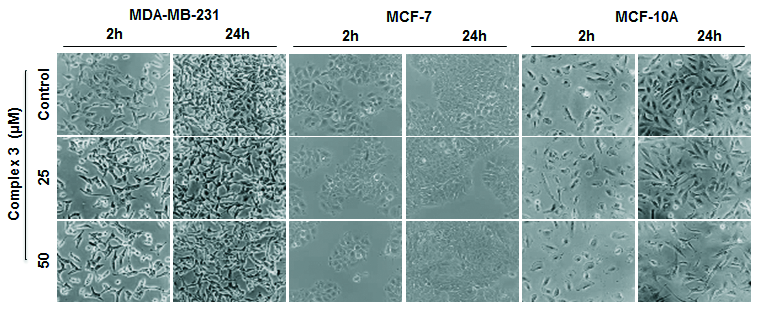

Supplement: S4 Fig — (TIF) [file pone.0183275.s006.tif]
